# Limited resources in clinical facilities: Experiences of nursing students during placements

**DOI:** 10.4102/curationis.v48i1.2732

**Published:** 2025-05-26

**Authors:** Julia L. Mafumo, Maria S. Maputle

**Affiliations:** 1 Department of Advanced Nursing Science, Faculty of Health Sciences, University of Venda, Thohoyandou, South Africa

**Keywords:** clinical learning, clinical supervision, healthcare facilities, limited resources, professional nurses, nursing student

## Abstract

**Background:**

Students need resources in the clinical areas to integrate theory and practice. When healthcare facilities have limited resources, students encounter momentous challenges that negatively affect their learning and supervision.

**Objectives:**

To explore the experiences of nursing students during placements in limited-resourced clinical facilities.

**Method:**

A qualitative approach with a phenomenological design was used. The setting was four hospitals, where students were placed for clinical experience. The population was nursing students in the third and fourth levels of their study who were sampled purposively. Data were collected through face-to-face semi-structured interviews and analysed using a coding method. Thematic analysis was done.

**Results:**

Limited staff led to poor student supervision and evaluation and absenteeism, and students were used as the working force, whereas limited resources led to the disintegration of theory and practice and procedures flawed.

**Conclusion:**

Resources in healthcare facilities are significant in student supervision. Therefore, the management in those facilities needs to ensure that the resources are always available.

**Contribution:**

The study contributes to bringing to light how the limited resources impact students’ learning and how this can impact future nursing practice.

## Introduction

Nursing students’ training has a component of theory and practice where students are placed in accredited healthcare facilities so that they can acquire skills and competencies. Healthcare facilities are clinical areas that afford nursing students the opportunity to develop critical thinking skills (Mafumo & Netshikweta 2022). When learners are in the clinical areas, they need the support of human and material resources so that they can perform the skills and be supervised and mentored. According to Mbakaya et al. (2022), clinical areas are vital in shaping the professional ethics and values of the students and encompass the environment and resources, which could be equipment, facilities, learning tools and standard procedures that influence students’ supervision. When clinical areas have limited resources, nursing students encounter momentous challenges that can affect their clinical learning and supervision (De Swardt 2019). Resources in clinical areas can be human, where there is a shortage of trained staff to supervise students, and material, where the shortage is of supplies and equipment used in the performance of skills. When students are allocated to the clinical learning areas, they are under the clinical supervision of professional nurses who guide them in clinical practice. Clinical supervision is a formal process of professional support and learning, enabling practitioners and students to develop knowledge and competence, assume responsibility for their own practice and enhance the protection of consumers and the safety of patients in clinical situations (Masamha et al. 2022).

Boniol et al. (2022) indicated that the World Health Organization (WHO 2020) has projected that by 2030, there will be a global shortage of 18 million healthcare workers. This has been noticed in the past two decades whereby globally there has been a noticeable shortage of nursing staff within the health sector (Boamah, Callen & Cruz 2021). The shortage of nursing staff was even exacerbated by the coronavirus disease 2019 (COVID-19) pandemic, where the number of patients in the hospitals increased with many nurses losing their lives, and others were left impaired, leading to a decreased workforce in the nursing fraternity (Lopez et al. 2022). The WHO and the International Council of Nurses (ICN) alluded that there is a global nursing shortage of 5.9 million nurses, whereby the greatest need is in Southeast Asia and Africa (Roth et al. 2022). The shortage of trained nurses directly impacts the clinical supervision of students. The responsibilities of professional nurses in the units include patient care, unit management and administration; teaching students and junior staff; and promoting research in the units (Meyer, Naude & Shangase 2009); therefore, the shortage of professional nurses has a direct impact on the teaching and training of nursing students. Galletta et al. (2017) and Gemuhay et al. (2019) argued that when there is a shortage of professional nurses in the clinical learning areas, students are used as the workforce, leading to them not focusing on their learning responsibilities but being caught up in being workers rather than students.

In the healthcare sector, a lot has been said about the reduced budget allocations leading to a shortage of supplies and equipment. When students are admitted to the nursing programme, they are provided with the simulation of procedures that are done in the clinical learning areas. Most of these simulation laboratories are in universities that are well-equipped with the necessary supplies and equipment. When students go to the clinical learning areas where there is a shortage of such, it leads to confusion among the students (Halcomb 2018). Many studies (Mulaudzi et al. 2020; Shamsi & Peyravi 2020; Tamata & Mohammadnezhad 2023) confirmed the challenges faced by healthcare workers in poorly resourced clinical facilities; however, in this article, the researchers aimed to explore the impact of the limited human and material resources in healthcare facilities on students’ clinical supervision in Limpopo province and further discuss the consequences for the education of nursing students.

### Problem statement

The training of nursing students constitutes theory and practice. The theory is provided by the nursing education institution. Before students are allocated to the clinical areas for practice, the nursing education institution provides students with practical exposure through simulation in the laboratories. Most of these simulation laboratories are well-equipped with the resources for teaching the procedures. On the contrary, many healthcare facilities are faced with challenges of shortage of resources, both human (Tamata & Mohammadnezhad 2023) and material (Modisakeng et al. 2020). The shortage of resources impacts the training of students, as indicated by those who stated that due to the shortage of supplies, some nurses were concerned about losing the information and skills they learnt in nursing schools and universities. When student nurses come back from clinical placements, they are expected to have reflective engagements with the lecturers. They often report that when they are allocated to these clinical facilities and are to perform these procedures, they find that there are no resources, leading to amazement and frustration. The researcher also finds that when students were to be assessed, there were inadequate resources, leading to a compromise of students’ training. Professional nurses are custodians of clinical supervision of students during placement. Due to the shortage of these nursing personnel, the responsibility to supervise is compromised. Most studies describe the impact of shortage of resources on clinical practice; it is in this light that the researcher needed to explore the consequences of limited resources on the learning of students.

### Purpose

The study sought to determine the experiences of student nurses related to limited resources in healthcare facilities in Limpopo province, South Africa.

## Research methods and design

The study was a qualitative approach with a hermeneutic phenomenological design to describe the impact of limited resources in healthcare facilities on the clinical supervision of nursing students. The lived experiences of participants are the main emphasis of hermeneutic phenomenology. It highlights the unique perspectives that people have within a specific setting (Suddick et al. 2020). The qualitative approach provided the opportunity for the participants to narrate in their own words and understand the experiences they had regarding limited resources in the clinical areas. The participants explained their experiences that limited resources in healthcare facilities had on their clinical supervision.

### Setting

The setting was four sampled hospitals in Vhembe and Capricorn districts of Limpopo province. Limpopo province is predominately located in the northern part of Limpopo. It has an estimated population of 6.1 million people (Statistics SA 2022). The province borders Zimbabwe in the north and Botswana in the west. The most spoken languages are Tshivenda, Xitsonga and Sepedi. The first sampled clinical facility is a tertiary hospital that offers more advanced care and is a referral for regional and district hospitals. The second one is a regional hospital that offers intermediate care to patients referred from the district hospitals and primary healthcare facilities. The remaining two hospitals are at the district level. Patients in these facilities are referred from the primary healthcare facilities. All these facilities are accredited by the South African Nursing Council to offer clinical learning for nursing students’ undergraduate programmes.

### Sampling of institutions

Four hospitals in the province where student nurses were training for the undergraduate nursing programme were allocated for clinical learning and were purposively sampled. The sampled hospitals were selected on the basis that they were accredited to be clinical learning areas for student nurses. In each district, two clinical facilities at different levels of care were selected. In one district, a tertiary and district hospital were selected; in the other, a regional and a district hospital were selected. The aim was to obtain information from different levels of healthcare, as these institutions have different budgets and staff allocations depending on the healthcare level. The hospitals had the largest number of students allocated in the two districts. It is for this reason that the researcher assumed that the more the number of learners the more information will be obtained (Mafumo, Luhallma & Maputle 2024). To ensure anonymity, each sampled hospital was coded. The codings thereof were AA, BB, CC and DD.

### Sampling of participants

Nonprobability purposive sampling was used to sample nursing students who were in the fourth and third levels of their study. The two levels of study were prioritised, as they were assumed to have more experience in clinical placement and would be able to provide relevant information. The inclusion criteria were that students should be registered in the undergraduate programme, be in the third or fourth level of study and be allocated to the sampled clinical areas. Both male and female student nurses were part of the study.

### Data collection

Data were collected through face-to-face semi-structured interviews at the four sampled clinical facilities. The researcher was the main research instrument that collected data. Student nurses who were interested in participation were recruited with the permission of the nursing education institutions and the nursing service managers in the institutions where they were allocated. In the first meeting, with the willing participants, the researcher introduced herself to the participants and the purpose and significance of the study were explained. Participants gave informed written consent before data were collected. Data were collected for a period of 7 months, from May 2019 to November 2019. Data were collected in a private room where there were no disturbances and privacy was ensured. The researcher used an interview guide. The following questions were asked of the participants:

1. Since you were allocated in this clinical area, do you or did you experience any limitation of staff and resources?
2. How did/does this impact your learning?

The researcher went on to probe participants to give clarity on some points given. Descriptive field notes were taken to document the date, time, setting and behaviours of participants during interviews, which were later used to support data analysis (Phillippi & Lauderdale 2018). A voice recorder was used to capture the narrative data, and permission for its use was first obtained from participants. The voice recorder was used so that the researcher could listen and transcribe data. The interviews were done in private rooms where there were no disturbances; the rooms were provided by the managers in the institutions. The comfort of the participants was ensured. Interviews were conducted in English as participants were fluent in the language. Data were collected until saturation was reached by participant number 25; however, the researcher interviewed four more participants to affirm that indeed data saturation was reached. Each interview lasted for 45 min – 55 min.

### Data analysis

Tesch’s (1990) open coding method was used to analyse data. The ideas that belonged together were grouped together to form themes and subthemes. During data analysis, the researcher started by listening to the voice recorder and reading through all the transcripts to get acquainted with the data collected and to jot down some ideas that came to mind. Thematic analysis was done. Topics that were similar were clustered and coded. After coding, data were reduced and examined closely for similarities and differences. Data that were similar were grouped into themes and subthemes. Data were analysed by two researchers who later compared and integrated the findings.

### Measures to ensure trustworthiness

To ensure trustworthiness, Lincoln and Guba’s (1985) four general criteria in qualitative research – credibility, transferability, dependability and confirmability – were used. To ensure credibility, the researcher used prolonged engagement where data were collected over a period of 7 months, allowing more time to be spent with the participants to explore the consequences that they faced due to the limited staff and equipment in the clinical learning areas. After data were collected, the researcher went to do member checking with the participants of one sampled institution to confirm if the findings were indeed what the participants indicated. In addition, the researcher interviewed four more participants after data saturation to affirm that, indeed, data saturation was reached. Transferability was ensured through a proper sampling of the participants and the collection of data using interviews. Dependability was ensured through detailed recording of the research process followed before and during data collection. Data were analysed by two people to ensure confirmability (Ahmed 2024).

### Demographic profile of participants

The participants in this study were predominantly female. In the 25 interviews conducted, 20 of the interviewees were females, whilst five were males. The participants were of the ages of 20–25 years. Twelve were in level three, and 13 were in level four of training. Two of the participants were repeating level four. The demographic profile for participants is indicated in Table 1.

**TABLE 1:** Participants’ demographic profile.

Participant	Age (years)	Level of study	Gender	Institution allocated
A	20	Three	Female	AA
B	22	Three	Female	AA
C	23	Three	Male	AA
D	20	Three	Female	AA
E	20	Three	Female	AA
F	23	Three	Male	AA
G	21	Three	Female	BB
H	22	Three	Female	BB
I	25	Three	Female	BB
J	21	Three	Female	BB
K	22	Three	Female	BB
L	24	Four (Repeating)	Female	BB
M	23	Four	Female	CC
N	23	Four	Female	CC
O	24	Four	Male	CC
P	23	Four	Female	CC
Q	25	Four	Male	CC
R	23	Four (Repeating)	Female	CC
S	24	Four	Female	DD
T	24	Four	Male	DD
U	23	Four	Female	DD
V	23	Four	Female	DD
W	25	Four	Female	DD
X	24	Four	Female	DD
Y	24	Four	Female	DD

AA, District hospital 1; BB, District hospital 2; CC, Regional hospital; DD, Tertiary hospital.

### Ethical considerations

The researcher followed the institutional processes for application to conduct research. Ethical clearance was obtained from the University of Venda Research Ethics Committee (reference no.: SHS/19/PGC/04/1103); thereafter, letters requesting permission to conduct the study were sent to the responsible structures and institutions involved. Ethical principles were adhered to. To ensure justice, participants were treated equally without any prejudice or bias; for autonomy, participants were explained in detail about the right to participate or not in the study. The issue of withdrawing from the study without any penalty was explained. Patients were not exposed to harm in any manner, as the interviews were held in a safe and private place. In order to protect the identity of participants and institutions, their names were not recorded, but the alphabets were used to identify the participants and institutions. Willing participants signed a consent form.

## Results

The two major themes that emerged were the shortage of staff and supplies and equipment in the clinical learning areas. The shortages affected the clinical supervision of students in different ways and were raised by a significant number of participants. The themes and subthemes are indicated in Table 2.

**TABLE 2:** Themes and subthemes.

Theme	Subtheme
1. The experiences related to the shortage of professional nurses	1.1 Inadequate supervision of students 1.2 Students used as a working force 1.3 Insufficient practical learning opportunities 1.4 Absenteeism
2. The experiences related to the shortage of equipment and supplies	2.1 Procedures are flawed 2.2 Failure to integrate theory and practice

### Theme 1: The experiences related to the shortage of professional nurses

Professional nurses are custodians of student learning in clinical areas. The shortage of professional nurses in the clinical learning areas had the following experiences:

#### Subtheme 1.1: Inadequate supervision of students

Participants alluded that due to the limited number of nurses in the units, proper supervision of students was not adequate. They stated that most of the time they are found wondering and working unsupervised, as there are few professional nurses. Another issue raised was that nurses in the units are overwhelmed by the number of patients in the units; therefore, supervision of students is less of their priority. Student feedback is also not effectively done due to staff shortage; if it is done, it is usually rushed and disadvantageous to the student. The subtheme is supported by the transcript where participant D said:

‘Medical ward is a busy ward all the time. The nurses there are few and even as students we can see that the work they are doing is too much. As a student, they hardly recognise us and do not attend to us.’ (Female, 20 years of age, third level of study)

Researcher: Can you elaborate further when you say they don’t attend to you?

Participant D:

‘In most cases, they don’t attend to us because we do procedures on our own without the proper supervision of professional nurses. Most of the time they are busy with taking rounds and helping very ill patients.’ (Female, 20 years of age, third level of study)

Participant B said:

‘In this unit where I am allocated, there is a shortage of nurses. Most of the time the nurses are busy, and you can’t blame them. The way professional nurses are so busy, they don’t even have time to assess if we have met our learning objectives. Sometimes the feedback is given hastily on the last day of placement because most of the time they have patients to take care of.’ (Female, 22 years of age, third level of study)

#### Subtheme 1.2: Students used as a working force

Participants were not impressed by the manner in which they were treated by the clinical staff. They felt that they were used to remedy the shortage of nursing staff, and this impacted their learning as they were given straight shifts to cover the ward shortage. The subtheme is supported by the following transcript:

Participant H:

‘The main problem that I have experienced in the ward due to the shortage of staff is when students are used as the working force of the units. The professional nurses would ask you to assist in working straight shifts because they are short-staffed. We are used to mend the shortage in the units. Especially when you are in your final year, they also delegate you tasks that are to be done by professional nurses like taking doctors’ rounds. Most of the time we are requested to work extra hours which they promise to pay back when the ward is not busy.’ (Female, 22 years of age, third level of study)

Researcher: How does that impact your learning in the unit?

Participant H:

‘At times I have assignments that I came with from block and when I am tired it is not easy to study. Even if I try, I find myself sleeping because of tiredness.’ (Female, 22 years of age, third level of study)

#### Subtheme 1.3: Insufficient practical learning opportunities

Poorly staffed clinical learning areas may limit the students’ time to learn and interact with patients. When students are in the clinical learning areas, they need guidance and support from professional nurses to learn how to interact with patients. If there is a shortage of staff, students are sometimes found wandering in the clinical learning areas not knowing what to do. Insufficient practical learning opportunities might hamper students’ ability to develop critical thinking skills, adaptability and resourcefulness as future nurses. The subtheme is supported by Participant B, who stated:

‘Professional nurses in the other wards are short-staffed and are always so busy that we don’t get time to ask them when we meet new conditions. They are always overwhelmed with the number of patients in such a way that students teaching is the least in their priorities., not that we blame them, but honestly, they don’t have time for students.’ (Female, 22 years of age, third level of study)

Participant L:

‘I am allocated in the surgical ward. The ward is so busy with only three professional nurses. On theatre days I find myself asking about the procedures that are to be done but really, the professional nurses will only answer in one sentence as they are busy with patients and need to finish leading to me hanging in the balance.’ (Female, 24 years of age, repeating fourth level of study)

#### Subtheme 1.4: Absenteeism

Absenteeism in nursing students has negative consequences. Students shared that sometimes they are absent from clinical placement because of discouragement and tiredness.

Participant L said:

‘The issue of students working long shifts makes us tired. In the ward where I was allocated previously, I was failing to cope and ended up being absent for two days.’ (Female, 24 years of age, repeating fourth level of study)

Researcher: What was it that you were not coping with?

‘The ward was so busy that I never had time to do my other personal things. The experience was so overwhelming when I had to be running around the whole day. I had two 12 hour shift and at the end of the second shift, I was physically and emotionally exhausted and I just needed a break.’ (Female, 24 years of age, repeating fourth level of study)

### Theme 2: The experiences related to the shortage of equipment and supplies

Students were concerned about the shortage of equipment and supplies in the clinical areas which impacted their professional socialisation. Insufficient equipment and supplies directly implicated their learning as theory and practice were disintegrated.

#### Subtheme 2.1: Procedures are flawed

Students alluded that due to the shortage of equipment and supplies, some procedures are not done as is done at the nursing education institutions’ simulation laboratories where they have adequate supplies and equipment. They indicated that in the clinical learning areas, there is a shortage of supplies, and therefore, procedures are flawed to accommodate what they have at that time. In support of the subtheme, Participant S said:

‘The challenge that I have regarding the shortage of equipment is that in the clinical learning areas, there are no resources like towels and when we do bed baths of patients, we don’t know what to use. In the University they tell us to use towels, but they are not there in clinical areas. When the lecturers come to evaluate you and when there is no equipment, it disadvantages me as a student.’ (Female, 24 years of age, fourth-level of study)

Participant C said:

‘Due to the shortage of equipment, most procedures are done not like what our lecturers taught us at school. Usually, nurses use what is available as long as it can do the work. At times aseptic technique is not adhered to when there is no sterile equipment.’ (Male, 23 years of age, third level of study)

#### Subtheme 2.2: Failure to integrate theory and practice

Students stated that the shortage of equipment affected how procedures in the nursing education institution and clinical learning areas are done. Simulation laboratories are usually equipped, whereas the clinical learning areas are not. This was supported by Participant M who said:

‘The challenge that I experienced is that in the University, they teach us procedures differently than how they are done in the hospitals. In the wards, we are usually in a hurry in order to assist patients and do not follow all the steps that are indicated in the book. When you are doing the assessments, you do it according to how you do it in the ward and this makes us fail.’ (Female, 20 years of age, fourth level of study)

Participant C said:

‘In the hospitals there are no equipment and supplies at times. There was a time when there were no urine bags in another hospital, gloves were attached to the catheter to collect urine. As a student, when I have to do intake and output it becomes difficult as the estimates are used other than the correct measurements.’ (Male, 23 years of age, third level of study)

## Discussion

The study aimed to determine the experiences of student nurses regarding limited resources in healthcare facilities on the clinical supervision of students. Clinical placements provide students with the opportunity to connect theory to practice as they get the opportunity to apply their acquired knowledge in real-life situations (Happell et al. 2020). Ideally, the number of staff and supplies in the healthcare facilities should be adequate so that the staff can provide patient care as well as offer support and supervision to nursing students during professional socialisation. A shortage of both equipment and supplies has negative experiences on the learning and professional socialisation of students. When students are in the clinical learning areas, they depend on the support and clinical supervision of professional nurses. According to the Nursing Education standards, professional nurses are expected to support students in the learning areas through clinical supervision so that they are competent, independent practitioners (*Nursing Act 33 2005*).

The shortage of trained nurses has been a concern in the last decade, especially in developing countries (Roth et al. 2022). The shortage affects both nursing education and practice. Professional nurses in the units are not only responsible for the clinical supervision of students, but they are also expected to balance the responsibilities of student supervision and patient care, and in most cases, patient care is the priority (Leonardsen et al. 2021). The situation is also exacerbated when there is a shortage of staff, as most of the time will be spent on patient care. An adequate number of professional nurses that match the number of students allocated in the units can promote effective supervision of students. However, this is not always the case, especially in developing countries where there is a shortage of nursing staff. Students are used to covering the shortages in the clinical areas. This sentiment is shared by Gemuhay et al. (2019), who stated that in instances where there is a shortage of staff, students are used to covering the shortage and are given the same shifts as working staff. When students are given huge responsibilities that drain them physically and emotionally at the expense of their learning. They become exhausted from working every day, affecting their physical and emotional well-being and leading to anxiety (Casafont et al. 2021).

In instances where students are used as the workforce, they absent themselves, as they don’t want to be treated as a workforce with long shifts (Magobolo & Dube 2019). Absenteeism has an impact on the training of students, as it affects the regulatory body has a prescribed number of hours that students are expected to acquire before the qualification is issued (Act 33 of 2005). It also impacts the future of nursing as a profession because the student who is continuously absent from clinical practice will not finish training on record time, whilst others might abscond from training. The amalgamation of limited supervision, increased workload and insufficient learning opportunities can lead to high levels of stress and burnout in students. When the stress level is chronic, it can negatively impact the students’ mental and physical well-being, compromising their learning experience and potential career choices (Gomathi, Jasmindebora & Baba 2017).

When students are performing tasks without the supervision of professional nurses, there are dangers of putting patients at risk, as students are still learning, and their competency is not yet guaranteed. This may also lead to students being unhappy, as argued by Mathe, Downing and Kearns (2021), who state that when student nurses are left to do work that they have limited knowledge about without support which they are not ready for, they feel used, and this can demoralise the student. In facilities where students are not adequately supported, they are also not given feedback on their performance, leading them to be unsure if they have achieved their learning objectives or not (Farzi, Shahriari & Farzi 2018).

The other limited resource that impacts nursing students’ clinical supervision is the lack of equipment in the clinical learning areas. This resulted in procedures not being done, as the students were taught in the simulation laboratories, disintegrating theory and practice. Integration of theory and practice in nursing education is crucial, as one is the basis for the other. Limited resources affecting students’ integration of theoretical knowledge and practical experience in the clinical setting can be a hindrance in the learning process, leading to fear, lack of confidence and anxiousness among students (Günay & Kılınç 2018; Farzi et al. 2018; Jahanpour et al. 2016). The study by Moyimane et al. (2017) concluded that in the district levels of care, the hospital experienced a severe scarcity of medical equipment, which manifested as a lack of equipment or as poor quality and poor maintenance of the few that were available. Nursing care, the nursing profession including student training and the hospital were all adversely affected by the shortage.

### Limitations

This study was conducted in the predominantly rural Limpopo province, where often there are limited resources of staff and resources. In urban settings, though they have limited resources, findings may yield different results. The study population was learner nurses registered for the undergraduate programme; however, postgraduate learners could have yielded different results.

### Recommendations

#### Recommendations for nursing education

Learning in the clinical areas can be stressful and intimidating to students, especially when students fail to integrate theory into practice. Nurse educators need to frequently do clinical accompaniment of students to assess the issues regarding students’ learning and, where possible, give assistance. The Nursing Education Institutions (NEIs) can also appoint preceptors who will be with the students every time they are in the clinical learning areas. The preceptors will provide students with guidance and support during clinical supervision.

The Department of Health has the responsibility to ascertain that there are adequate staff and resources in the healthcare facilities. This is to ensure that there is quality patient care and student learning and practice. The management of hospitals should understand that limited resources do not only impact patient care but student learning as well. Vacant posts should be filled, and the budget should be allocated according to the institutional and unit needs. The budgets should be reviewed and adjusted accordingly.

#### Recommendations for nursing research

The clinical learning areas provide students with skills and clinical practice. It is in these areas where competency is attained. In the absence of guidance and adequate support, it might be difficult to achieve competency of students. Research of mitigating factors that leads to not filling vacant post and limited resources should be carried out so that such can be prevented.

## Conclusion

Poorly staffed clinical learning areas negatively impact students’ clinical supervision, affecting their educational and professional growth. Limited supervision, increased workload, inadequate experiential learning opportunities, reduced collaboration and increased stress levels are some of the consequences of limited resources. Addressing these limitations requires collaborative efforts from nursing education institutions, healthcare facilities and policymakers to prioritise adequate staffing, support systems and quality learning environments. By recognising and addressing these challenges, we can ensure nursing students receive the necessary resources and experiences to become competent, compassionate and resilient nurses in the future.
